# The Newly Sequenced Genome of *Pisum sativum* Is Replete with Potential G-Quadruplex-Forming Sequences—Implications for Evolution and Biological Regulation

**DOI:** 10.3390/ijms23158482

**Published:** 2022-07-30

**Authors:** Michaela Dobrovolná, Natália Bohálová, Vratislav Peška, Jiawei Wang, Yu Luo, Martin Bartas, Adriana Volná, Jean-Louis Mergny, Václav Brázda

**Affiliations:** 1 Institute of Biophysics of the Czech Academy of Sciences, 612 65 Brno, Czech Republic; dobrovolna@ibp.cz (M.D.); natalia.bohalova@ibp.cz (N.B.); vpeska@ibp.cz (V.P.); 2 Faculty of Chemistry, Brno University of Technology, Purkyňova 118, 612 00 Brno, Czech Republic; 3 Department of Experimental Biology, Faculty of Science, Masaryk University, 611 37 Brno, Czech Republic; 4 Laboratoire d’Optique et Biosciences (LOB), Ecole Polytechnique, CNRS, INSERM, Institut Polytechnique de Paris, CEDEX, 91128 Palaiseau, France; jiawei.wang@polytechnique.edu (J.W.); yu.luo@curie.fr (Y.L.); 5 CNRS UMR9187, INSERM U1196, Université Paris-Saclay, CEDEX, 91405 Orsay, France; 6 Department of Biology and Ecology, Faculty of Science, University of Ostrava, 710 00 Ostrava, Czech Republic; martin.bartas@osu.cz; 7 Department of Physics, Faculty of Science, University of Ostrava, 710 00 Ostrava, Czech Republic; adriana.volna@osu.cz

**Keywords:** G-quadruplex, G4 propensity, chloroplast DNA, sequence prediction

## Abstract

G-quadruplexes (G4s) have been long considered rare and physiologically unimportant in vitro curiosities, but recent methodological advances have proved their presence and functions in vivo. Moreover, in addition to their functional relevance in bacteria and animals, including humans, their importance has been recently demonstrated in evolutionarily distinct plant species. In this study, we analyzed the genome of *Pisum sativum* (garden pea, or the so-called green pea), a unique member of the *Fabaceae* family. Our results showed that this genome contained putative G4 sequences (PQSs). Interestingly, these PQSs were located nonrandomly in the nuclear genome. We also found PQSs in mitochondrial (mt) and chloroplast (cp) DNA, and we experimentally confirmed G4 formation for sequences found in these two organelles. The frequency of PQSs for nuclear DNA was 0.42 PQSs per thousand base pairs (kbp), in the same range as for cpDNA (0.53/kbp), but significantly lower than what was found for mitochondrial DNA (1.58/kbp). In the nuclear genome, PQSs were mainly associated with regulatory regions, including 5′UTRs, and upstream of the rRNA region. In contrast to genomic DNA, PQSs were located around RNA genes in cpDNA and mtDNA. Interestingly, PQSs were also associated with specific transposable elements such as TIR and LTR and around them, pointing to their role in their spreading in nuclear DNA. The nonrandom localization of PQSs uncovered their evolutionary and functional significance in the *Pisum sativum* genome.

## 1. Introduction

*Pisum sativum*, commonly known as the garden pea or green pea, is an important and broadly cultivated crop worldwide. It was domesticated ~10,000 years ago in the Near East [1]. Its seeds are rich in proteins, fibers, vitamins, minerals, and antioxidants [2]. In addition, the pea is widely used as a model plant species nowadays [3,4], and also is a historically important genetic model as the first organism for which the basic genetics laws were described and demonstrated by the Moravian monk Gregor Johann Mendel in 1865 [5,6]. His systematic work, statistic evaluation, and mathematical descriptions of his experiments with hereditary of seven independent pea features paved the foundation of modern genetics. His discoveries were later called the laws of Mendelian inheritance in his honor.

G-quadruplexes (G4s) are four-stranded DNA or RNA structures in which alternative Hoogsteen base pairing (G-G) enables guanine tetrad formation. Each guanine tetrad corresponds to one stack of G4 structure and is stabilized by an internal spine of positively charged ions, mostly sodium (Na^+^) or potassium (K^+^). Depending on the number of guanine tetrads (stacks) we can distinguish two-, three-, four-, five-, or even six-stacked G4s [7]. As described above, G4 formation requires guanines—at least eight for a two-tetrad structure—and is thus favored in regions locally enriched in this nucleotide. From a functional perspective, G4s have been documented to influence replication, transcription, and even translation, which illustrates their importance in basic physiological cellular processes and indicates the need for their precise regulation [8,9,10]. A recent study showed the distinct roles of G4 in the transcription regulation of the rice genome based on its genomic localization. G4 found in promoters had a potentized effect, whereas gene location caused repression of gene transcription [11].

The presence of G4s has been demonstrated in viral [12,13], bacterial [14], archaeal [15], fungal [16,17], and other eukaryotic genomes, including that of humans [18]. However, only a few genome-wide analyses of G4s in plants have been reported [11,19,20,21,22]. Genome-wide analyses of G4s have not been reported for *P. sativum,* the genome of which was sequenced and assembled 3 years ago [23]. *P. sativum* nuclear genome is composed of two metacentric and five acrocentric chromosomes [24]. The pea genome is relatively large (4.45 Gb) compared to other *Fabaceae*, such as *Glycine max* (soybean)—995 Mb, *Medicago truncatula* (barrel medic)—(420 Mb, or *Lotus japonicus* (bird’s-foot trefoil)—385 Mb, mostly due to genome expansion of transposons, which comprise about 76% of the pea genome [25]. Several analysts suggested faster evolution of the pea genome in comparison with the species mentioned above due to frequent recombination events mediated by transposons [23,26]. Here, we performed analyses of the presence and localization of PQSs in the *P. sativum* genome, including its linear nuclear chromosomes and its mitochondrial (mt) and chloroplast (cp) DNA, and we found experimental evidence that sequences found in these two organelles adopted G4 structures in vitro.

## 2. Results

### 2.1. Comparison of PQS Sequences in P. sativum Genome

The fully sequenced genome of *P. sativum* in the NCBI database consists of seven chromosomes, mitochondrial DNA (mtDNA), and chloroplast DNA (cpDNA). The length of *P. sativum* chromosomes varies between 372 Mbp for chromosome I and 580 Mbp for chromosome V. *P. sativum* mtDNA is 363,843 bp long and cpDNA is 122,035 bp long. G4Hunter analyses with standard values for G4Hunter (i.e., a window size of 25 nucleotides and threshold score of 1.2), showed over 1.3 million PQSs in *P. sativum* genome (Table 1).

**Figure 1:**
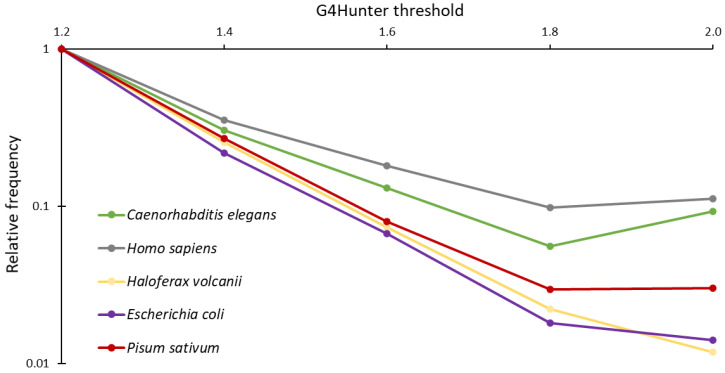
Comparison of G4Hunter score distribution across the different phylogenetic groups. Note the stronger counterselection against high-stability G4s in prokaryotes. *P. sativum*, with an initial slope closer to prokaryotes than to the two other eukaryotes studied here, exhibited an increase in PQS frequency with the highest analyzed G4Hunter score.

In total, we found 1,355,394 PQSs, with no obvious strand bias (679,713 in one strand and 675,681 in the complementary strand). Detailed results for each sequence are presented in the Supplementary Materials (Table S1). As expected, the most abundant PQSs had a moderate G4Hunter score (G4HS) in the 1.2–1.4 category (70.1% of all PQSs), followed by sequences in the 1.4–1.6 (19.2% of all PQSs) and 1.6–1.8 (5.7% of all PQSs) intervals. Sequences with a high G4HS (1.8–2.0 interval: 28,513 PQSs; 2.0–more: 28,801 PQSs) were the least frequent. As expected, the number of PQSs tended to decrease with the G4Hunter threshold. The frequency of the PQSs with the G4Hunter score in the 1.2–1.6 interval was higher in mtDNA than in nuclear DNA and cpDNA. We compared the distribution of G4HSs in *P. sativum* with the relative frequencies of PQSs in various organisms (Figure 1). While the genome of *P. sativum* contained more PQSs with a G4HS above 1.8 and 2.0 compared to prokaryotic genomes of *Escherichia coli* (bacteria) and *Haloferax volcanii* (Archaea), the number of PQSs in these categories was higher in animals such as *C. elegans* and *H. sapiens*.

We then performed analyses using GC content as an additional parameter to evaluate the influence of GC content on PQS density. The average GC content in the nuclear DNA was 30.02%, with a minimum of 29.68% for chromosome II and a maximum of 31.07% for chromosome I. The frequency of PQSs per 1000 GC for genomic DNA was 1.395. The highest GC content (45.07%) and the highest number of PQSs per 1000 GC (3.494) were found in mtDNA. Chloroplast DNA contained 1.531 PQSs per 1000 GC pairs, with a GC content of 34.78%. However, the frequency of PQSs in chromosomal DNA was very similar for all chromosomes, and varied between 0.411 PQS per kbp for chromosome II and 0.432 PQS per kbp for chromosome I, with an average of 0.419 PQS/kbp. The frequency of PQSs in cpDNA was 0.533 PQS/kbp (i.e., slightly higher than in nuclear DNA), while the highest PQS frequency was found in mtDNA (1.575 PQSs per 1000 nucleotides, corresponding to 573 PQSs in a 364 kbp genome). In other words, the density in PQSs was nearly four times higher in mtDNA than in nuclear DNA. The total PQS counts and the percentage of GC and PQS frequency characteristics for each sequence are summarized in Table 2.

### 2.2. Experimental Demonstration of G4 Formation for Pisum mtDNA and cpDNA Sequences

**Figure 2:**
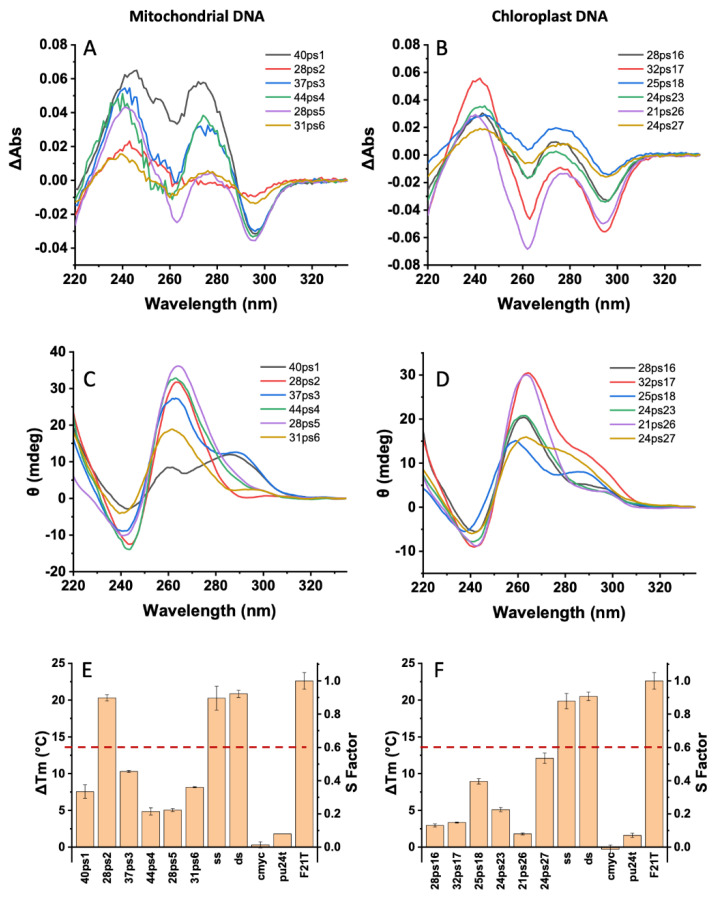
Experimental evidence for G4 formation. (**A**,**B**) Isothermal difference spectra (IDS); (**C**,**D**) circular dichroism spectra; (**E**,**F**) FRET-MC results for the mitochondrial (**left**) and chloroplast (**right**) sequences. In panels E and F, ss and ds correspond to single- and double-stranded negative controls, while cmyc and pu24t are G4-forming positive controls. F21T corresponds to the delta Tm observed in the absence of any competitor (S = 1). The red dotted line corresponds to the threshold under which a sequence was considered to form a quadruplex [15,28].

Among the 573 mtDNA and 65 cpDNA PQSs, we chose 12 candidate sequences (six mtDNA and six cpDNA) spanning G4H scores between 1.25 and 2.0 (Table 3) and representative of motifs found in these organelles. We used a combination of biophysical methods to confirm G4 formation in vitro, as illustrated in Figure 2. As inferred from isothermal difference spectra (IDS) (Figure 2A,B), circular dichroism (CD) spectra (Figure 2C,D), and FRET-MC (Figure 2E,F) for most (11/12) motifs clearly formed G4s at room temperature, while some ambiguity remained for 28ps2. Of note, the majority of spectra (8 out of 12) suggested a parallel fold. This bias was the result of relatively high G4Hunter scores (average G4H = 1.61) and the fact that we systematically introduced non-G nucleotides at both extremities, as flanking nucleotides favor a parallel topology [27].

### 2.3. Localization of PQSs in P. sativum Genome

**Figure 3:**
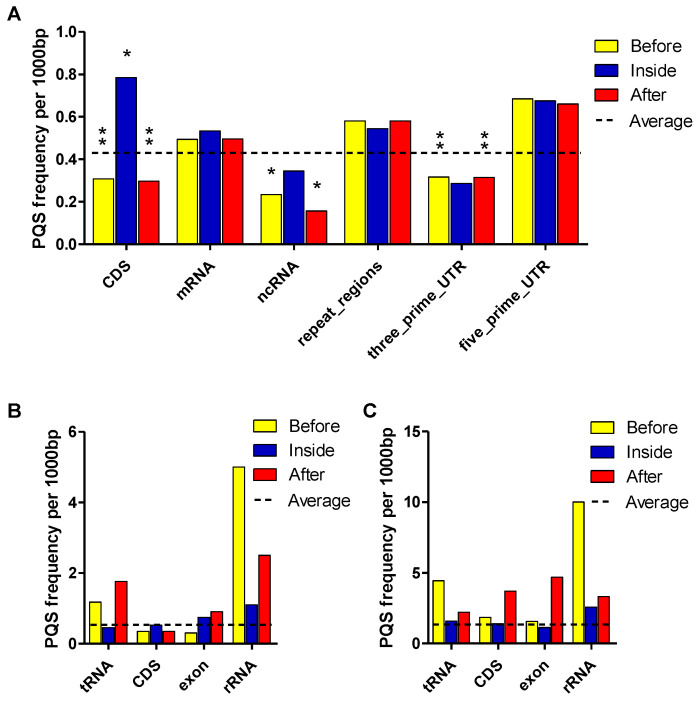
Differences in PQS frequency according to DNA locus. The chart shows PQS frequencies normalized per 1000 bp annotated locations from the NCBI database. We analyzed the frequencies of all PQSs within (inside), before (100 bp), and after (100 bp) annotated locations in (**A**) genomic DNA, (**B**) mtDNA, and (**C**) cpDNA. Dashed lines denote the average PQS frequency in corresponding DNA. Statistical significance of annotated locations in genomic DNA was related to the average chromosomal PQS frequencies according to a Kruskal–Wallis test, followed by Dunn’s pairwise comparison with Bonferroni correction of the *p*-value. Asterisks denote statistical significance: * *p*-value < 0.05; ** *p*-value < 0.01.

To analyze the localization of PQSs in *P. sativum*, we downloaded annotations from the NCBI genome database and overlayed the PQS presence with described *features* and repeats identified de novo in RepeatExplorer2. In addition to the direct presence within the *features,* we also analyzed the presence of PQSs 100 bp before and after the *feature* annotations (Figure 3; Table S2 in the Supplementary Materials). An analysis of PQSs in annotated features and repeats showed that the distribution of PQSs throughout the genome was not uniform. The highest frequency of PQSs per kbp in genomic DNA was found within coding regions (CDS (0.785)), and around *repeat regions* ((0.580)—retrotransposons and transposons) within the *mRNA* (0.534). A notable enrichment in PQSs was also found within 5′UTR, while few PQSs were present in 3′UTR. The lowest PQS frequency was found before or after *ncRNA* (0.23 and 0.16) and *within* 3′UTR (0.287). The density around 3′UTR (0.315) was also lower than average. The annotations for genomic DNA are shown in Figure 3A. The telomeric motif of *P. sativum* has been known for a long time [29]; however, the telomeres were not annotated in the current assembly. The telomeric repeats of *P. sativum* were composed of TTTAGGG repeats, which had a G4Hunter score of 1.29. G4 formation with this motif has been previously demonstrated [30,31], and the stability of the corresponding G4 was relatively high (Tm = 64 °C in 100mM KCl; nearly as high as the human hexanucleotide GGGTTA motif). There were ≈142 TTTAGGG motifs per kbp of telomeric DNA, which would allow the formation of up to 35–36 G4s per kbp, but the formation of multiple juxtaposed G4s has not been experimentally investigated for this plant motif.

In mtDNA, the highest PQS frequencies per kbp were found within 100 bp after exon (4.688), before tRNA (4.444), and after CDS (3.703), followed by region 100 bp after (3.333) and inside rRNA (2.575). The most notable enrichment of PQSs was found in the regions before rRNA, where the PQS frequency per 1000 bp reached 10. The lowest PQS frequency was found within the exon region (1.130). Differences in PQS frequency according to annotated features in mtDNA are shown in Figure 3B.

In cpDNA, a high PQS frequency within features was also observed 100 bp before (5.0) and after (2.5) rRNA, similarly to mtDNA. The frequency inside rRNA was almost 5-times lower than the frequency before this feature (see Figure 3C).

### 2.4. PQSs in Transposable Elements

**Figure 4:**
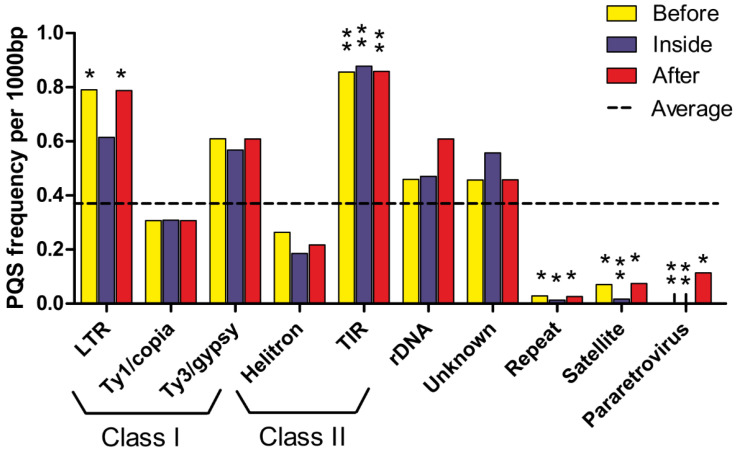
Differences in PQS frequency by repeat region. The chart shows PQS frequencies normalized per 1000 bp of annotated transposons. We analyzed the frequencies of all PQSs within (inside), before (100 bp), and after (100 bp) annotated transposons in genomic DNA. The dashed line denotes the average PQS frequency in transposons. Statistical significance is shown as in Figure 3. *p* < 0.05, * *p* < 0.01, ** *p* < 0.001.

Using the G4Hunter algorithm, we analyzed *P. sativum* repeat regions to determine the frequency and distribution of PQSs in transposable elements (TEs). In the case of the *P. sativum* genome, TEs represented over 80%, with a significant contribution by Ogre elements, which is a group of LTR retrotransposons (Class I) [32]. LTR retrotransposons of the superfamilies Ty3-gypsy and Ty1-copia were the dominant group, with over 91% and over 8.5% of the LTR sequence coverage, respectively. The transposons (Class II) represented a smaller part of the genome. Over 99% of all Class II transposons were terminal-inverted repeat (TIR) transposons, and less than 1% were helitrons. Satellite and ribosomal DNA (rDNA) formed a small fraction of all annotated TEs. Short tandem repeats annotated as Pararetrovirus were the result of the viral sequence integration [33]. When only total PQSs were considered, the largest number of PQSs in annotated transposons was found within Ty3/gypsy, Ty1/copia, and 100 bp before/after Ty3/gypsy. To evaluate the localization of PQSs within TEs, we overlapped PQSs with annotated locations and analyzed the frequencies of all PQSs within, 100 bp before, and 100 bp after annotated TEs (Figure 4). The only TEs with a higher frequency of PQSs inside than before and after were unclassified transposons and Ty1-copia.

The highest PQS frequency per kbp was observed within TIR transposons. High PQS frequencies were found before and after unclassified LTRs (almost 1.5-times more frequent than in gene regions). Repeats and satellites had the lowest PQS frequencies per kbp, and their PQS frequencies compared to the gene region were 5 times less frequent than in gene regions. No PQSs were found at 100 bp before and inside Pararetrovirus. However, this was not the same when considering PQS frequency per kbp after Pararetrovirus. The data are available in Table S4 in the Supplementary Materials.

## 3. Discussion

The recent improvements in the sequencing methods of and computational approaches to full-genome analyses allowed effective searches for PQSs. The G4Hunter algorithm was successfully used to select PQSs with a high probability of G4 structure formation and minimum positive or false negative results in various genomes from viruses, bacteria, and eukaryotes, including the human genome.

However, the number of plant genomes analyzed for G4 propensity is still limited, and is mostly performed using older pattern-based algorithms. There are several reasons for this: the plant genomes are usually huge, and compared to an animal, there are not so many fully assembled plant genomes; moreover, the number of repetitive sequences in some plant genomes is enormous, and these repetitive sequences are challenging for the correct assembly in the genomes. Genomic DNA from *P. sativum* contained various repetitive sequences involving transposable elements (TE). Previous studies showed that the TE fraction represented a significant portion of plant genomes, and could vary from 15 to 30% in *Arabidopsis thaliana* (thale cress) and *Brachypodium distachyon* (purple false brome), and from 70 to 80% in species such as *Zea mays* ssp. *mays* (maize) and *Hordeum vulgare* (barley) [34]. In the case of the *P. sativum* genome, TEs represent more than 80% [32]. Therefore, we took advantage of the contemporary sequenced genome of *P. sativum* and performed G4Hunter analyses to determine the presence and localization of PQSs within classic features, as well as TEs. PQSs have been identified in various plant genomes, including *A. thaliana*, *Oryza sativa* subsp. *Japonica* (rice), *Populus trichocarpa* (black cottonwood), and *Vitis vinifera* (common grape) [35]. Previous pattern-based PQS analyses (Quadparser G3L1-7; corresponding to a motif involving four runs of at least three guanines separated by loops of one to seven nucleotides) demonstrated that Arabidopsis had only 9 G4 motifs/Mbp, while rice had 92 G4 motifs/Mbp, a 10-fold difference, and the monocot plant sample (barley, maize, and rice) had a higher PQS frequency compared to dicots (soybean, common grapevine, and *Arabidopsis thaliana*) [21,36,37]. It is hard to compare various algorithms for PQS prediction; however, considering only PQSs with a G4Hunter score above 1.4, which represents a very stable G4 as evaluated in vitro, the frequency of PQSs in *P. sativum* genome seemed higher than within previously reported dicot plants.

However, the more interesting aspect was the huge difference in PQSs between nuclear and organelle DNA, especially mtDNA. While the frequency of PQSs in all chromosomes was similar (around 0.41 per kbp), mtDNA had more than five times as many PQSs, suggesting a different regulation for *P. sativum* linear nuclear and circular mtDNA. PQSs also had different localizations in mtDNA compared to PQS localization in nuclear DNA; therefore, we suggest that G4-formation and regulatory pathways differ in circular and linear DNAs. Interestingly the comparison of mtDNA PQS frequencies among various species showed an increased PQS frequency for vertebrates as well as for land plants, contrary to a lower PQS frequency in the mtDNA of protists and fungi [38]. In animals, it has been shown that G4s play a direct role in mitochondrial genome replication, transcription processivity, and respiratory function [39]. The significantly higher frequency of PQSs in *P. sativum* mtDNA compared to those in nuclear DNA suggested that this observation may also be valid for plant mitochondria. We analyzed a dozen sequences in vitro that were extracted from *P. sativum* mitochondrial (mt) and chloroplast (cp) DNA, and provided experimental evidence that the motifs found in these two organelles were prone to G4 formation in vitro. This study constitutes, to the best of our knowledge, the first experimental evidence that chloroplast sequences may form G4s.

Generally, it is accepted that very stable G4s tend to be strongly counter-selected, and low-scoring PQSs (with a G4HS between 1.2 and 1.6) tend to constitute the vast majority of G4-prone motifs. The main reason is probably the high stability of G4s formed by PQSs with a G4HS > 1.8, which therefore constitutes a physical barrier for most biological processes such as replication or transcription [40]. Interestingly, there was no significant drop in PQS density in the *P. sativum* genome for high G4H scores. This was in contrast with what has been observed in most other species, as previous analyses revealed that most of the PQSs found in Platyhelminthes [41], Archaea [15], and bacteria [14] have a relatively low G4Hunter score, and the number of PQSs in these organisms decreases sharply above a score of 1.6. Strikingly, nuclear DNA and mt- and cpDNA differ not only in PQS frequencies, but also in the localization of these PQSs. For circular cp- and mtDNA, there was a strong abundance around RNA genes, while in genomic DNA, there was a significant difference in PQS frequency for 3′UTR, where PQS were more than twice as less frequent inside compared to 5′UTR. The 5′UTR serves as the binding point for the ribosome, which allows the ribosome to bind and initiate translation [42]. The higher density of PQSs in this region suggested important regulatory roles of G4 motifs in the process of translation. Many G4-binding proteins in animals and humans are known [43]. Recently, it has been shown that proteins in barley seedlings can bind to PQSs and form DNA–protein complexes [44], so we can expect that G4-binding proteins also will be present in plant genomes [45].

In conclusion, we analyzed the presence of PQSs in cpDNA and TE for the first time. The nonrandom localization of PQSs in the genome of *P. sativum* suggested their regulatory function and the importance of LTR and TIR transposons. This supported the hypothesis that TEs may serve as vehicles for the genomic spread of G4s [46]. In addition, the higher density of PQSs in mtDNA and cpDNA compared to regular chromosomes suggested specific roles for quadruplexes in organelles.

## 4. Materials and Methods

### 4.1. Process of Analysis

The complete DNA sequences of the *P. sativum* genome, including nuclear, mt, and cp genomes were downloaded (20 June 2021) in FASTA format from the National Center for Biotechnology Information (NCBI) [47]. NCBI IDs are listed in Table S1 in the Supplementary Materials. For putative PQS prediction, the new and strengthened computational core of our DNA analyzer software written in Java programming language was used [48]. For our analyses, we used an actualized G4Hunter algorithm implementation [49] with default parameters for G4Hunter—a window size of 25 and a G4Hunter score (G4HS) above 1.2 (the chosen value of 25 nucleotides corresponded to the size of a typical intramolecular G4). The default values for G4Hunter have been previously discussed and validated [50]. G4HSs were then grouped in five intervals: 1.2 up to 1.4, 1.4 up to 1.6, 1.6 up to 1.8, 1.8 up to 2.0, and 2.0 and more. Data were merged in a single Excel file (accessible in Table S1 in the Supplementary Materials) for further analyses and statistical evaluations.

### 4.2. Analysis of Repetitive DNA from Unassembled Reads Using RepeatExplorer2 and TAREAN

Only the conserved coding domains of the repeats were annotated in the available genome assembly [23]. Therefore, we performed an independent de novo identification of repeats and annotated genomic loci corresponding to them, including their specific regions such as LTRs, spacer sequences, etc. We used publicly available low-pass whole-genome sequencing data in FASTQ format from the Sequence Read Archive of the NCBI (Run ERR063464) [51]. We performed standard preprocessing, a quality check, and interlacing of paired-end reads, and ran RepeatExplorer2 and TAREAN analyses with 2,913,990 reads of a uniform length of 100 nt [52]. The results were manually checked, and the sequences of selected repeats—mobile elements (LTR, Ty1/copia, Ty3/gypsy, TIR, helitron, pararetrovirus), satellites, rDNA, and unclassified repeats—were used in BLAST against the pea genome for the purpose of repeat loci annotation according to the feature table (see below) completion.

### 4.3. Sequence Matching and Transposon Annotation (BLAST)

The BLAST database was constructed from the pea genomic sequence (accessible in Table S1 in the Supplementary Materials), and the sequences from our RepeatExplorer2 analysis (see Section 2.2) were used as a query in blastn with parameters as follows: -outfmt 6-max_target_seqs 10000000-num_threads 4-evalue 0.1. The blast match positions were then used for the feature table completion.

### 4.4. Analysis of PQSs around Annotated NCBI Features and Repeats from Our RepeatExplorer2 Analysis

The feature table containing functional annotations of the *P. sativum* genome was downloaded from the NCBI database. Features describe the functions and locations of sequences within the genome of an organism [53]. We performed an analysis of PQS occurrence inside uploaded features, as well as 100 bp before and after each feature. Features were grouped by their name stated in the feature table file (gene, rRNA, tRNA, ncRNA, and repeat region). Further processing was performed in Microsoft Excel, and the resulting data are available in Table S2 in the Supplementary Materials.

### 4.5. Statistical Analysis

Outliers were detected using the function chisq.out.test from the outliers package in R version 4.0.5 [54]. Normal distribution of PQS frequencies in annotated locations was determined using the Shapiro–Wilk test, and statistical significance was evaluated using the nonparametric Kruskal–Wallis test. Multiple pairwise comparisons were assessed using a post hoc Dunn’s test with Bonferroni correction of the significance level.

### 4.6. Experimental Demonstration of G4 Formation

DNA sequence matching motifs found in Pisum chloroplast and mitochondrial DNA were synthesized by Eurogentec (Seraing, Belgium) and used without further purification. Concentrations were determined using the extinction coefficients provided by the manufacturer. Isothermal difference spectra (IDS) and circular dichroism (CD) spectra were recorded as previously described [28]. FRET-MC provided a convenient independent method to detect G4 formation; detailed experimental protocols can be found in [15,28]. Briefly, in this test, G4-forming competitors led to a marked decrease in the ligand-induced stabilization effect (∆*T*_m_), while nonspecific competitors (e.g., single- or double-stranded sequences) had little effect.

## Figures and Tables

**Table 1:** Total number and frequencies of PQSs found in *P. sativum* genome grouped according to G4Hunter score (1.2–1.4 means any sequence with a score between 1.2 and 1.399; 1.4–1.6 between 1.4 and 1.599, etc.).

G4Hunter Threshold	Number of PQSs	PQS Frequency (PQS/kbp)
	**Genomic DNA**	
1.2–1.4	960,462	0.30
1.4–1.6	260,428	0.081
1.6–1.8	76,552	0.024
1.8–2.0	28,513	0.0088
2.0–more	28,801	0.0089
	**mtDNA**	
1.2–1.4	377	1.04
1.4–1.6	117	0.32
1.6–1.8	47	0.13
1.8–2.0	16	0.044
2.0–more	16	0.044
	**cpDNA**	
1.2–1.4	40	0.33
1.4–1.6	15	0.12
1.6–1.8	8	0.066
1.8–2.0	1	0.0082
2.0–more	1	0.0082

**Table 2:** The overall number of PQSs found with a G4Hunter score of 1.2 or above; their frequencies per kbp; GC content; length of all PQSs (all base pairs with potential to form G4) divided by the total number of bp in the DNA (PQSs); and the number of PQSs per thousand GC for each chromosome, mtDNA, and cpDNA.

DNA Sequence	Length (Mb)	Number of PQS	PQS Frequency (/kbp)	GC Content (%)	PQSs (%)	PQSs/GC%
Chr I	372.17	160,922	0.432	31.07	1.31	1.392
Chr II	427.60	175,744	0.411	29.68	1.24	1.385
Chr III	437.56	181,878	0.416	29.72	1.26	1.399
Chr IV	446.35	184,737	0.414	29.90	1.25	1.384
Chr V	579.27	244,737	0.422	30.13	1.28	1.402
Chr VI	480.42	200,963	0.418	29.81	1.27	1.403
Chr VII	491.38	205,775	0.419	29.87	1.27	1.402
*Total nuclear*	3234.74	1,354,756	0.419	30.02	1.27	1.395
mtDNA	0.36	573	**1.575**	45.07	4.81	3.494
cpDNA	0.12	65	0.533	34.78	1.65	1.531

**Table 3:** Twelve sequences were analyzed using three different biophysical methods (IDS: isothermal difference spectra; CD: circular dichroism; FRET-MC, a competition fluorescence melting assay). G4Hunter score is indicated in the column labeled “G4H”. Concl. column indicates the conclusion reached based on these three methods. “+” stands for positive, meaning that the method indicated the sequence was forming a G4.

*Name*	*Sequence*	*G4H*	*IDS*	*CD*	*FRET-MC*	*Concl.*
**Mitochondrial sequences:**						
40ps1	TGGGCGTCTGGGGTTGGTTTAAGGAAAAATCGGGGTCGGA	1.25	+	+	+	G4
28ps2	AGGGATCAAGAAACGGATAGGGAGGGGA	1.32	?	+	-	G4?
37ps3	AGGGAGGACCGGGGGCCAGAGCAAGTTGGGTTGGGGT	1.41	+	+	+	G4
44ps4	TGGGGCGAGGGTCTTTCATTAAAGGGGGGAAAAGAGGGGTGGGT	1.66	+	+	+	G4
28ps5	CGGGGGCGGGTTCTGAGCAGGATGGGGA	1.68	+	+	+	G4
31ps6	AGGAAGCGGGGGGAGGAACACAGGGGAAGGA	1.61	+	+	+	G4
**Chloroplast sequences:**						
28ps16	TGGAAGGGGTCAATAAGGGGTTGGGGGA	1.96	+	+	+	G4
32ps17	CGGGGGGTAGATTGGGGCGTGGACATAAGGGT	1.62	+	+	+	G4
25ps18	TGGGATCCGGGCGGTCCAGGGGGGA	1.48	+	?	+	G4
24ps23	AGGGGTGGGGACAGAGGTTTTGGT	1.67	+	+	+	G4
21ps26	TGGGGGTGGTGAAGGGAGGGC	2.00	+	+	+	G4
24ps27	CGGGGTGGAGACGATGGGGTCGGT	1.62	+	?	+	G4

## Data Availability

All data are available in the paper and supplementary materials.

## Supplementary Materials

The following supporting information can be downloaded at: https://www.mdpi.com/article/10.3390/ijms23158482/s1.
