# Supplementary material for: The Newly Sequenced Genome of Pisum sativum Is Replete with Potential G-Quadruplex-Forming Sequences—Implications for Evolution and Biological Regulation
Source: Int J Mol Sci. 2022 Jul 30;23(15):8482. doi: 10.3390/ijms23158482 (PMC9369095; doi:10.3390/ijms23158482)

Supplementary Material Table S1; G4Hunter results

Supplementary Material Table S1: Results; (A) the DNA sequence; (B) the NCBI accession numbers for genomic sequences; (C), assigned tags corresponding to the generic name of analyzed organism; (D) count of every nucleotide; (E) the total length of genome; (F) GC count of genome; (G) gc content in %; (H) the total count of PQS found; (I) the PQS frequency per 1,000 nt; (J) the number of PQS per 1,000 GC; (K-O) the the total count of PQS with different G4hunter score 1.2-1.4 interval; (P-T), the total lenght of PQSs with different G4Hunter score interval; (U) the total lenght of all PQS found; (V) total length of PQS divided by genome length ; (W-AA) shows the frequency of PQS with different G4Hunter score interval; (AB-AH) the coverage of the sequence by PQS in different G4Hunter score intervals. For every column with numerical data, there is statistical analyses below results.

A	B	C	D	E
**name**	**ncbi**	**tags**	**nucleic_count**	**length [bp]**
Chromosome_1LG6	LR535892.1	ntDNA1	{'T': 97427444, 'A': 97525019, 'G': 57822712, 'N': 61592043, 'C': 57797928}	372165146
Chromosome_2LG1	LR535893.1	ntDNA2	{'T': 104462357, 'A': 104614492, 'G': 63459355, 'N': 91625082, 'C': 63436632}	427597918
Chromosome_3LG5	LR535894.1	ntDNA3	{'T': 107542520, 'A': 107585623, 'G': 65045590, 'N': 92386042, 'C': 65001026}	437560801
Chromosome_4LG4	LR535895.1	ntDNA4	{'T': 111470141, 'A': 111475147, 'G': 66656269, 'N': 89966344, 'C': 66782776}	446350677
Chromosome_5LG3	LR535896.1	ntDNA5	{'T': 145534245, 'A': 145744379, 'G': 87333136, 'N': 113467812, 'C': 87189499}	579269071
Chromosome_6LG2	LR535897.1	ntDNA6	{'T': 119471705, 'A': 119510303, 'G': 71682987, 'N': 98221522, 'C': 71530532}	480417049
Chromosome_7LG7	LR535898.1	ntDNA7	{'T': 122118968, 'A': 122335647, 'G': 73411942, 'N': 100127006, 'C': 73387399}	491380962
Mitochondrial DNA	MW073102.1	mtDNA	{'A': 99980, 'G': 82270, 'C': 81732, 'T': 99861}	363843
Chloroplast DNA	MG882488	cpDNA	{'G': 20732, 'A': 39077, 'C': 21716, 'T': 40510}	122035

**STATISTICS**				
**SUM**				3235227502
**CNT**				9
**AVG**				359469722
**MIN**				122035
**MAX**				579269071
**VAR**				39653057699341000
**STDEV**				211210061
**Q1**				372165146
**MEDIAN Q2**				437560801
**Q3**				480417049

Supplementary Material Table S1; G4Hunter results

Supplementary Material Table S1: Results; (A) the DNA sequence; (B) the NCBI accession numbers for genomic sequences; (C), assigned tags corresponding to the generic name of analyzed organism; (D) count of every nucleotide; (E) the total length of genome; (F) GC count of genome; (G) gc content in %; (H) the total count of PQS found; (I) the PQS frequency per 1,000 nt; (J) the number of PQS per 1,000 GC; (K-O) the the total count of PQS with different G4hunter score 1.2-1.4 interval; (P-T), the total lenght of PQSs with different G4Hunter score interval; (U) the total lenght of all PQS found; (V) total length of PQS divided by genome length ; (W-AA) shows the frequency of PQS with different G4Hunter score interval; (AB-AH) the coverage of the sequence by PQS in different G4Hunter score intervals. For every column with numerical data, there is statistical analyses below results.

F	G	H	I	J	K	L	M
**gc_count [bp]**	**gc %**	**PQS result_count**	**PQS frequency**	**PQS/GC**	**result_count 1.2-1.4**	**result_count 1.4-1.6**	**result_count 1.6-1.8**
115620640	31.07	160922	0.432394064	1.39	157590	2762	349
126895987	29.68	175744	0.411002937	1.38	172448	2789	291
130046616	29.72	181878	0.415663377	1.40	178551	2824	286
133439045	29.90	184737	0.413883096	1.38	181180	2960	363
174522635	30.13	244737	0.42249278	1.40	240149	3847	419
143213519	29.81	200963	0.418309468	1.40	197072	3268	359
146799341	29.87	205775	0.418768768	1.40	201642	3468	408
164002	45.07	573	1.574855089	3.49	567	6	0
42448	34.78	65	0.53263408	1.53	64	1	0
970744233	290.03	1355394.00	5.04	14.79	1329263.00	21925.00	2475.00
9	9.00	9.00	9.00	9.00	9.00	9.00	9.00
107860470	32.23	150599.33	0.56	1.64	147695.89	2436.11	275.00
42448	29.68	65.00	0.41	1.38	64.00	1.00	0.00
174522635	45.07	244737.00	1.57	3.49	240149.00	3847.00	419.00
3555803644195100	22.99	6943754065.11	0.13	0.43	6680181065.21	1804255.43	23369.78
63247760	5.09	88383.95	0.38	0.70	86690.27	1424.71	162.14
115620640	29.81	160922.00	0.42	1.39	157590.00	2762.00	286.00
130046616	29.90	181878.00	0.42	1.40	178551.00	2824.00	349.00
143213519	31.07	200963.00	0.43	1.40	197072.00	3268.00	363.00

Supplementary Material Table S1; G4Hunter results

Supplementary Material Table S1: Results; (A) the DNA sequence; (B) the NCBI accession numbers for genomic sequences; (C), assigned tags corresponding to and the generic name of analyzed organism; (D) count of every nucleotide; (E) the total length of genome; (F) GC count of genome; (G) gc content in %; (H) the total count of PQS found; (I) the PQS frequency per 1,000 nt; (J) the number of PQS per 1,000 GC; (K-O) the the total count of PQS with different G4hunter score 1.2-1.4 interval; (P-T), the total lenght of PQSs with different G4Hunter score interval; (U) the total lenght of all PQS found; (V) total length of PQS divided by genome length ; (W-AA) shows the frequency of PQS with different G4Hunter score interval; (AB-AH) the coverage of the sequence by PQS in different G4Hunter score intervals. For every column with numerical data, there is statistical analyses below results.

N	O	P	Q	R	S	T	U
**result_count 1.8-2.0**	**result_count 2.0 and more**	**lenght 1.2-1,4**	**lenght 1.4-1.6**	**lenght 1.6-1.8**	**lenght 1.8-2.0**	**lenght 2.0 and more**	**lenght all**
105	116	4741532	102120	22055	8054	15507	4889268
97	119	5165179	99780	18598	8102	13250	5304909
94	123	5356445	99312	16675	7240	15729	5495401
110	124	5442049	105379	23661	8687	19565	5599341
157	165	7221541	139542	24433	11823	18890	7416229
136	128	5922792	119488	21770	10162	15814	6090026
113	144	6057336	126339	25974	9032	19458	6238139
0	0	17314	181	0	0	0	17495
0	0	1988	31	0	0	0	2019

812.00	919.00	39926176.00	792172.00	153166.00	63100.00	118213.00	41052827.00
9.00	9.00	9.00	9.00	9.00	9.00	9.00	9.00
90.22	102.11	4436241.78	88019.11	17018.44	7011.11	13134.78	4561425.22
0.00	0.00	1988.00	31.00	0.00	0.00	0.00	2019.00
157.00	165.00	7221541.00	139542.00	25974.00	11823.00	19565.00	7416229.00
2669.28	3180.77	6029796977884.84	2370755894.32	89901464.25	15624041.65	53227465.51	6371467770409.51
54.80	59.82	2604519.46	51643.98	10056.80	4192.50	7738.27	2677293.64
94.00	116.00	4741532.00	99312.00	16675.00	7240.00	13250.00	4889268.00
105.00	123.00	5356445.00	102120.00	21770.00	8102.00	15729.00	5495401.00
113.00	128.00	5922792.00	119488.00	23661.00	9032.00	18890.00	6090026.00

Supplementary Material Table S1; G4Hunter results

Supplementary Material Table S1: Results; (A) the DNA sequence; (B) the NCBI accession numbers for genomic sequences; (C), assigned tags corresponding to and the generic name of analyzed organism; (D) count of every nucleotide; (E) the total length of genome; (F) GC count of genome; (G) gc content in %; (H) the total count of PQS found; (I) the PQS frequency per 1,000 nt; (J) the number of PQS per 1,000 GC; (K-O) the the total count of PQS with different G4hunter score 1.2-1.4 interval; (P-T), the total lenght of PQSs with different G4Hunter score interval; (U) the total lenght of all PQS found; (V) total length of PQS divided by genome length ; (W-AA) shows the frequency of PQS with different G4Hunter score interval; (AB-AH) the coverage of the sequence by PQS in different G4Hunter score intervals. For every column with numerical data, there is statistical analyses below results.

V	W	X	Y	Z	AA
**lenght %**	**frequency 1.2-1.4**	**frequency 1.4-1.6**	**frequency 1.6-1.8**	**frequency 1.8-2.0**	**frequency 2.0 and more**
1.31	0.42344	0.00742	0.00094	0.00028	0.00031
1.24	0.40329	0.00652	0.00068	0.00023	0.00028
1.26	0.40806	0.00645	0.00065	0.00021	0.00028
1.25	0.40591	0.00663	0.00081	0.00025	0.00028
1.28	0.41457	0.00664	0.00072	0.00027	0.00028
1.27	0.41021	0.00680	0.00075	0.00028	0.00027
1.27	0.41036	0.00706	0.00083	0.00023	0.00029
4.81	1.55836	0.01649	0.00000	0.00000	0.00000
1.65	0.52444	0.00819	0.00000	0.00000	0.00000
15.35	4.958654	0.072216	0.005386	0.001754	0.001993
9.00	9.000000	9.000000	9.000000	9.000000	9.000000
1.71	0.550962	0.008024	0.000598	0.000195	0.000221
1.24	0.403295	0.006454	0.000000	0.000000	0.000000
4.81	1.558364	0.016491	0.000938	0.000283	0.000312
1.22	0.128141	0.000009	0.000000	0.000000	0.000000
1.17	0.379683	0.003222	0.000350	0.000113	0.000126
1.26	0.408060	0.006632	0.000654	0.000215	0.000266
1.27	0.410358	0.006802	0.000723	0.000230	0.000278
1.31	0.423441	0.007421	0.000813	0.000271	0.000285

Supplementary Material Table S1; G4Hunter results

Supplementary Material Table S1: Results; (A) the DNA sequence; (B) the NCBI accession numbers for genomic sequences; (C), assigned tags corresponding to and the generic name of analyzed organism; (D) count of every nucleotide; (E) the total length of genome; (F) GC count of genome; (G) gc content in %; (H) the total count of PQS found; (I) the PQS frequency per 1,000 nt; (J) the number of PQS per 1,000 GC; (K-O) the the total count of PQS with different G4hunter score 1.2-1.4 interval; (P-T), the total lenght of PQSs with different G4Hunter score interval; (U) the total lenght of all PQS found; (V) total length of PQS divided by genome length ; (W-AA) shows the frequency of PQS with different G4Hunter score interval; (AB-AH) the coverage of the sequence by PQS in different G4Hunter score intervals. For every column with numerical data, there is statistical analyses below results.

AB	AC	AD	AE	AF	AG	AH
**Coverage [nt] 1.2-1.4**	**Coverage [nt] 1.4-1.6**	**Coverage [nt] 1.6-1.8**	**Coverage [nt] 1.8-2.0**	**Coverage [nt] 2.0 and more**	**Coverage [nt] all**	**Coverage all [%]**
4058970	101513	21978	8262	15434	4170855	1.121
4437919	99223	18520	8220	13250	4540973	1.062
4598392	98874	16623	7498	15729	4701598	1.075
4676201	104935	23578	8687	19542	4796576	1.075
6193091	138979	24387	12042	18843	6339760	1.094
5086405	118871	21770	10620	15734	5211974	1.085
5194160	125561	25854	9234	19382	5329415	1.085
14243	181	0	0	0	14396	3.957
1805	31	0	0	0	1820	1.491

34261186.00	788168.00	152710.00	64563.00	117914.00	35107367.00	13.04
9.00	9.00	9.00	9.00	9.00	9.00	9.00
3806798.44	87574.22	16967.78	7173.67	13101.56	3900818.56	1.45
1805.00	31.00	0.00	0.00	0.00	1820.00	1.06
6193091.00	138979.00	25854.00	12042.00	19542.00	6339760.00	3.96
4440265989431.58	2347265660.40	89356415.28	16380055.11	52942729.80	4660140922061.36	0.80
2235016.61	51387.49	10026.26	4292.73	7717.55	2289685.25	0.95
4058970.00	98874.00	16623.00	7498.00	13250.00	4170855.00	1.07
4598392.00	101513.00	21770.00	8262.00	15729.00	4701598.00	1.08
5086405.00	118871.00	23578.00	9234.00	18843.00	5211974.00	1.12

Supplementary Material Table S2; Annotation results

Nuclear DNA												
	**PQS count**	**All before**	**All in**	**All after**	**PQS frequency per 1000bp**	**All before**	**All in**	**All after**	**PQS frequency ratio to gene**	**All before**	**All in**	**All after**
**Feature**	CDS	7222	40539	6969		0.308	0.785	0.297		0.589	1.341	0.558
	exon	7192	53823	6942		0.287	0.656	0.277		0.549	1.120	0.520
	five_prime_UTR	2633	7898	2541		0.685	0.675	0.661		1.310	1.154	1.241
	gene	2002	64115	2040		0.523	0.585	0.532		1.000	1.000	1.000
	mRNA	2478	89501	2484		0.494	0.534	0.495		0.946	0.912	0.930
	ncRNA	6	22	4		0.469	0.689	0.313		0.897	1.177	0.587
	repeat_regions	10310738	15682488	10297699		0.581	0.545	0.580		1.111	0.930	1.089
	three_prime_UTR	1173	5387	1163		0.317	0.287	0.315		0.607	0.490	0.591

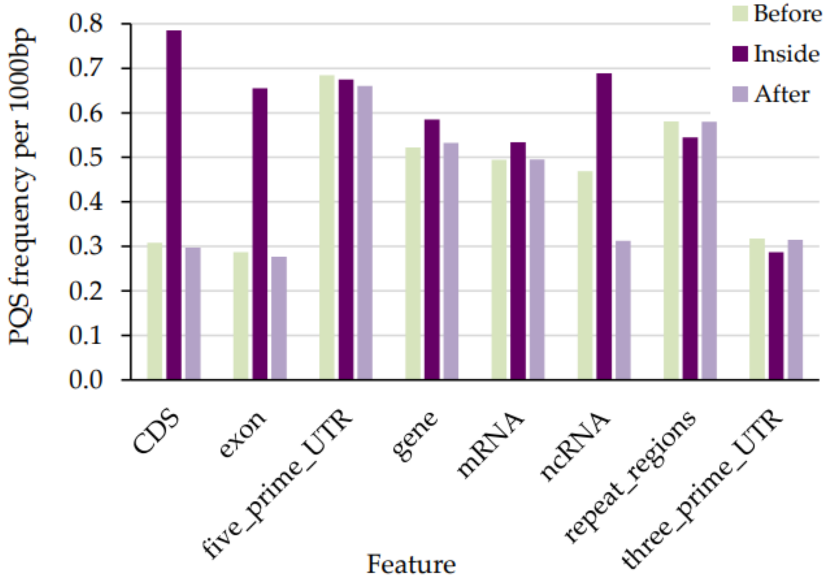

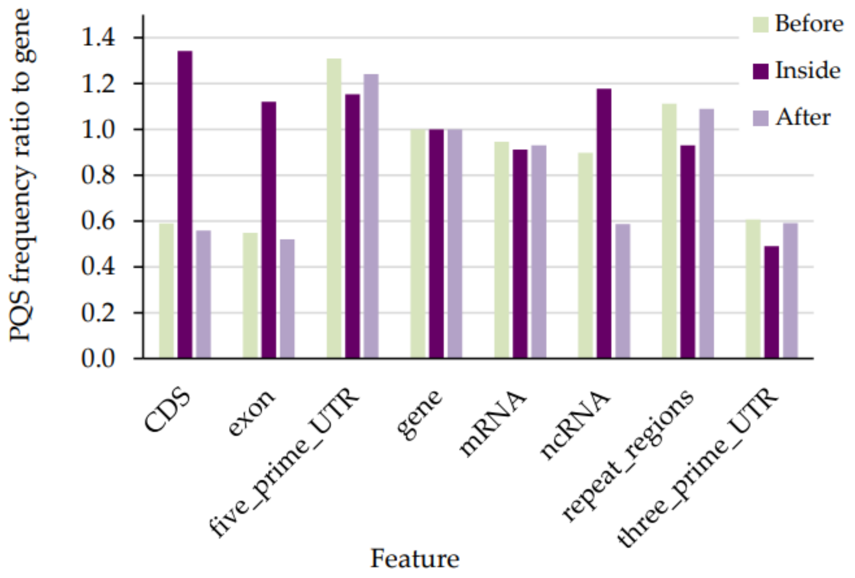

Supplementary Material Table S2; Annotation results

Mitochondrial DNA												
	PQS count	All before	All in	All after	PQS frequency per 1000bp	All before	All in	All after	PQS frequency ratio to gene	All before	All in	All after
Feature	gene	20	115	19		3.125	2.159	2.969		1.000	1.000	1.000
	tRNA	8	2	4		4.444	1.576	2.222		1.422	0.730	0.749
	CDS	10	42	20		1.852	1.408	3.704		0.593	0.652	1.248
	exon	5	12	15		1.563	1.130	4.688		0.500	0.524	1.579
	rRNA	3	13	1		10.000	2.575	3.333		3.200	1.193	1.123

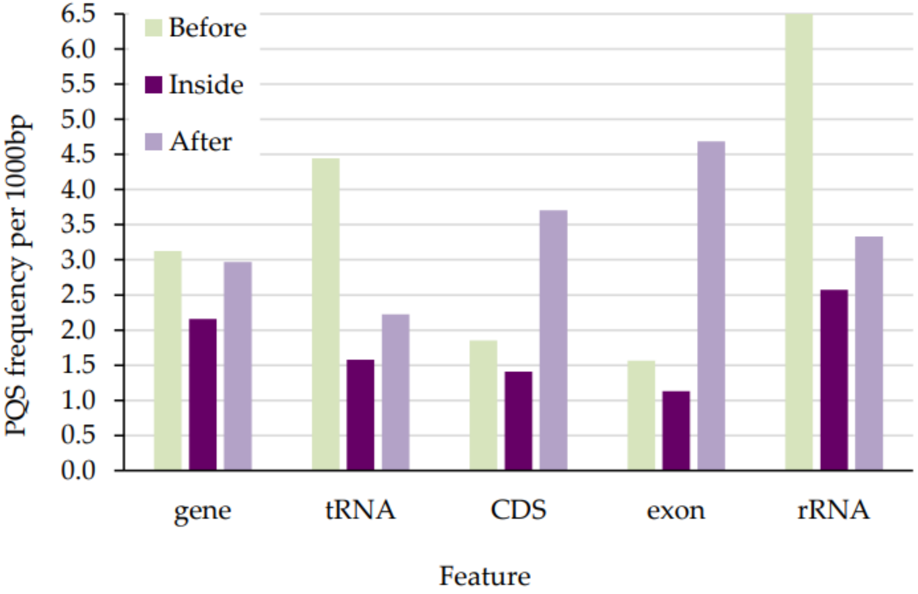

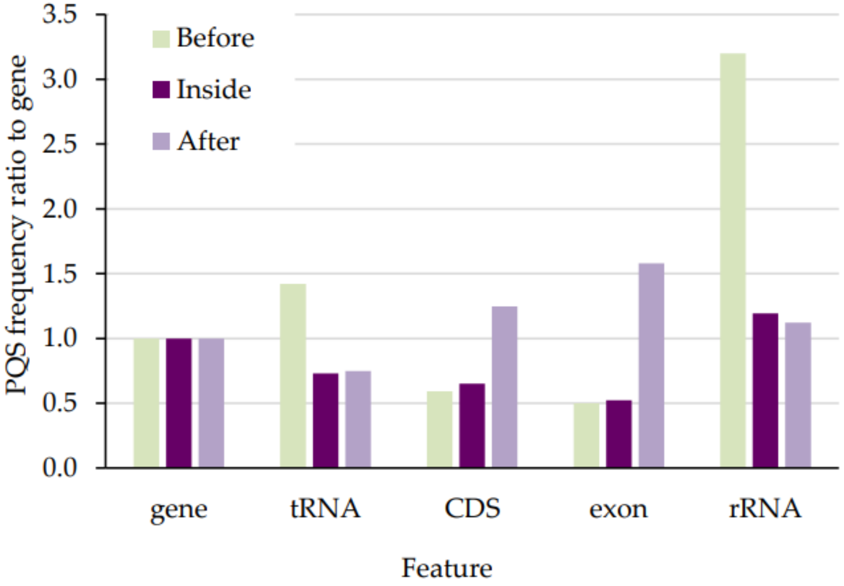

Supplementary Material Table S2; Annotation results

Chloroplast DNA												
	PQS count	All before	All in	All after	PQS frequency per 1000bp	All before	All in	All after	PQS frequency ratio to gene	All before	All in	All after
Feature	gene	9	44	9		0.826	0.511	0.826		1.000	1.000	1.000
	tRNA	4	1	6		1.176	0.454	1.765		1.425	0.888	2.137
	CDS	3	34	3		0.349	0.521	0.349		0.422	1.020	0.422
	exon	1	7	3		0.303	0.743	0.909		0.367	1.454	1.101
	rRNA	2	5	1		5.000	1.105	2.500		6.056	2.162	3.028

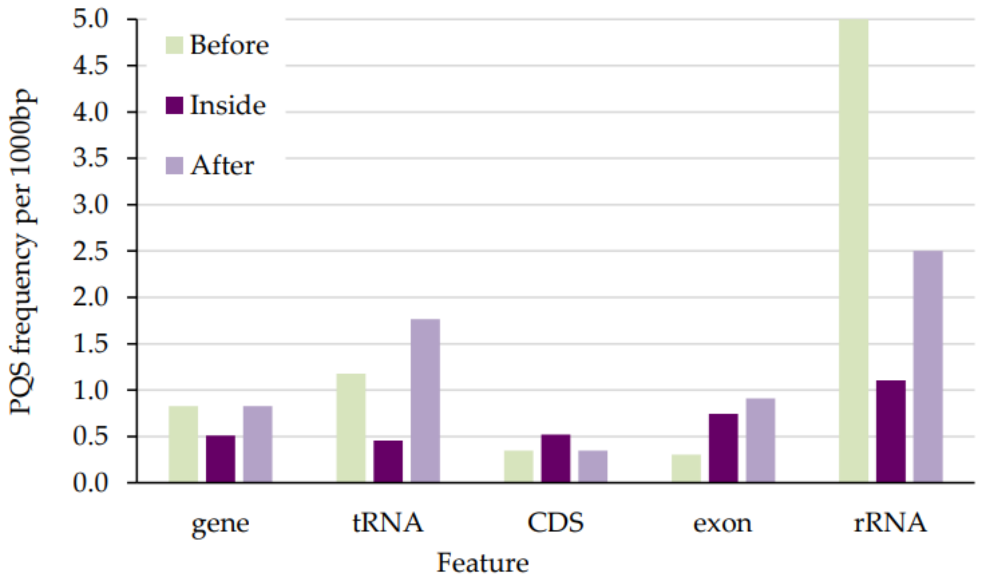

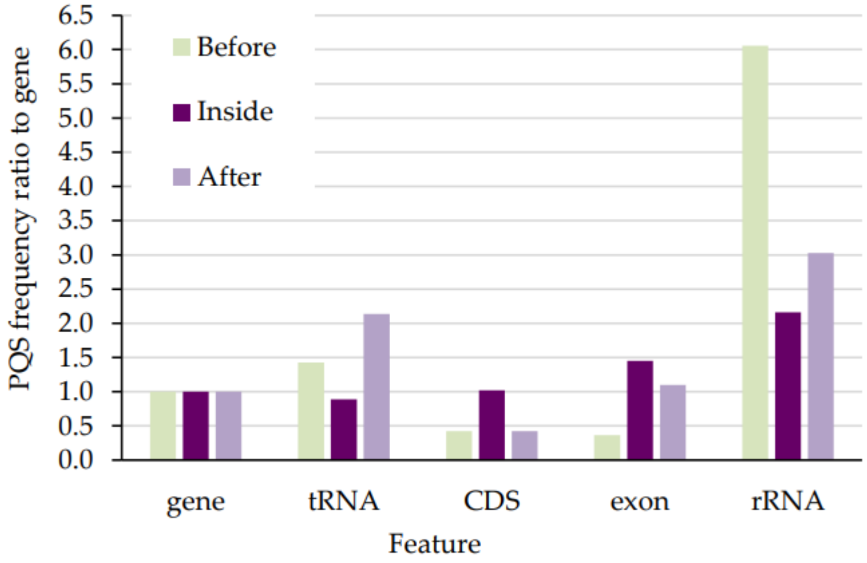

Supplementary Material Table S3; Total number of PQS found on sense and antisense DNA strand

Supplementary Table S1. Total number of PQS found on sense and antisense DNA strand - sumarized (A), nuclear DNA (B), mtDNA (C) and cpDNA (D)

A

**Number of PQS (sense strand)**	**Number of PQS (antisense strand)**
679713	675681

B

**Number of PQS (sense strand)**	**Number of PQS (antisense strand)**
679400	675356

C

**Number of PQS (sense strand)**	**Number of PQS (antisense strand)**
289	284

D

**Number of PQS (sense strand)**	**Number of PQS (antisense strand)**
24	41

Supplementary Material Table S4; Transposable elements annotation

Transposable elements											
	**PQS count**	**All before**	**All in**	**All after**	**PQS frequency per 1000bp**	**All before**	**All in**	**All after**	**PQS frequency ratio to gene**	**All before**	**All in**	**All after**
**Feature**	Helitron	148	254	122		0.264	0.185	0.217		0.504	0.316	0.408
	LTR	20400	25936	20354		0.790	0.615	0.788		1.512	1.050	1.480
	pararetrovirus	0	0	2		0.000	0.000	0.114		0.000	0.000	0.213
	plastid	193	409	233		0.691	0.400	0.834		1.321	0.684	1.566
	rDNA	92	242	122		0.459	0.470	0.609		0.879	0.803	1.144
	repeat	83	92	77		0.029	0.013	0.026		0.055	0.022	0.050
	satellite	6684	3166	7140		0.070	0.017	0.075		0.134	0.030	0.141
	TIR	64136	126740	64352		0.856	0.878	0.859		1.638	1.500	1.613
	Ty1/copia	443208	737370	442748		0.307	0.308	0.307		0.588	0.527	0.576
	Ty3/gypsy	9629685	14551992	9616201		0.610	0.568	0.609		1.167	0.971	1.144
	unknown	146109	236287	146348		0.457	0.557	0.458		0.874	0.951	0.859
	gene	2002	64115	2040		0.523	0.585	0.532		1.000	1.000	1.000

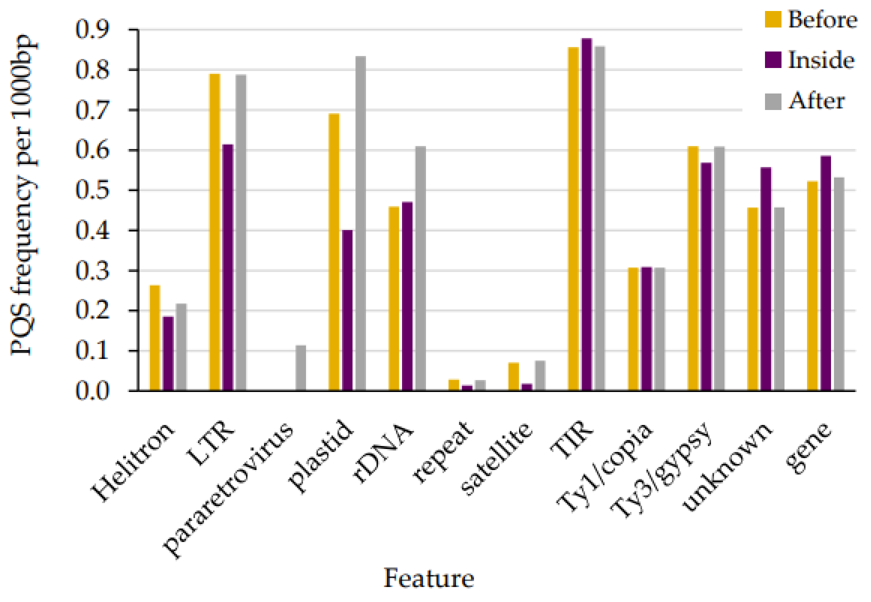

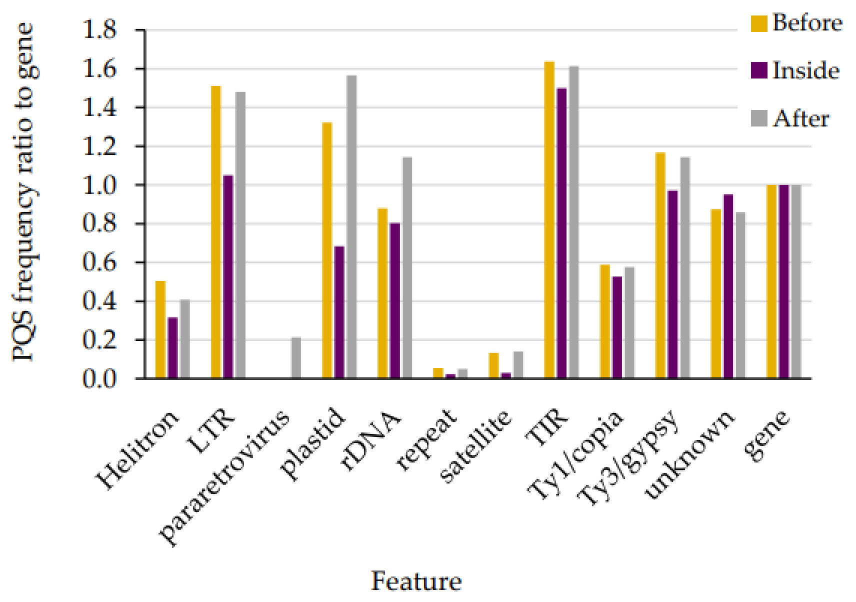

Supplement: Supplementary file 1 [file ijms-23-08482-s001.zip › ijms-1828585-supplementary.pdf]
